# Proteoforms as the true units of physiological function

**DOI:** 10.1007/s00421-025-06096-3

**Published:** 2025-12-19

**Authors:** James N. Cobley

**Affiliations:** The University of Dundee, Dundee, Scotland, UK

**Keywords:** Proteoforms, Phenotypes, Proteomics, Cysteine, Redox

## Abstract

Proteomics has matured into a discipline capable of quantifying nearly every protein encoded by the genome, yet it remains largely blind to the true operational units of physiology: proteoforms. Each proteoform—defined by a specific sequence and post-translationally modified state—represents a unique molecular identity with distinct chemical, functional, and structural properties. This review proposes the *proteoform functor*: a mathematical map between the abstract proteoform state space and the realised physiological space of biological function—and ultimately complex phenotypes. This mapping is not linear or additive. Rather, it is hierarchical, nonlinear, and context-dependent, reflecting the emergent complexity of life. Without resolving proteoforms, proteomics risks describing shadows of biology rather than its material substance. Deciphering complex phenotypes, demands a shift from bulk protein averages to revealing the precise molecular identities—proteoforms—that give rise to physiology.

## Introduction

Imagine trying to open a set of locks, each requiring a unique combinatorial access code, that remains elusive. Deciphering the code is difficult: For just ten possible digits and ten locks, the number of possible combinations balloons to $\:{10}^{10}$, ten billion, possibilities.

By analogy, unravelling the protein-basis of complex phenotypes, such as neurodegenerative diseases (Halliwell 2006; Cobley 2018; Cobley et al. 2018; Sidlauskaite et al. 2018; Li et al. 2025; Sarnataro et al. 2025; Stavrovskaya et al. 2025), requires one to decipher the molecular identity of the instantiated protein molecules out of a possible $\:>\:1\:\times\:{10}^{20}$, one hundred quintillion, proteoforms. This staggering combinatorial landscape defines the grand challenge of modern proteomics: to move beyond bulk averages to reveal the precise molecular identities that give rise to physiology (Burnum-Johnson et al. 2022).

Despite their centrality to biological function, proteoforms—the distinct molecular species arising from combinations of post-translational modifications, sequence variants, truncations, and conformational states—remain largely invisible in most proteomic analyses (Smith et al. 2013). Current workflows collapse these discrete biochemical states into aggregate signals, effectively averaging across functionally distinct populations. This abstraction severely limits the information that can be derived from even the most comprehensive datasets.

For example, an increase in total protein abundance may mask the fact that the relevant phenotype is driven by a small, transient population of a *trebly phosphorylated proteoform*. Such combinatorial states often evade detection even in PTM-focused studies, which typically quantify modifications site-by-site rather than as integrated molecular identities. Resultantly, the link between molecular mechanism and physiological outcome is blurred, producing substantial knowledge gaps in understanding how proteins operate in living systems (Ball 2025).

This work aims to clarify why proteoform-level resolution is not merely a technical refinement but a conceptual and physiological necessity. Using cysteine proteoforms—the operational units of redox biology—as illustrative examples (Cobley 2025a), this work reviews how modern analytical, computational, and theoretical advances can transform proteomics into a *proteoform-resolved science*. Framing these developments within the concept of the *proteoform functor*, provides a unifying model. One that connects sequence, state, and function through a continuous informational mapping with the goal of understanding how their distribution, dynamics, and interactions give rise to the emergent properties of physiology.

The actionable take-home message is simple: Even small-scale, proteoform-level analyses using existing technologies can provide vital information on the proteoform functor, connecting molecular biology and physiology.

## Proteoforms: unique information-carrying molecular entities

**Fig. 1:**
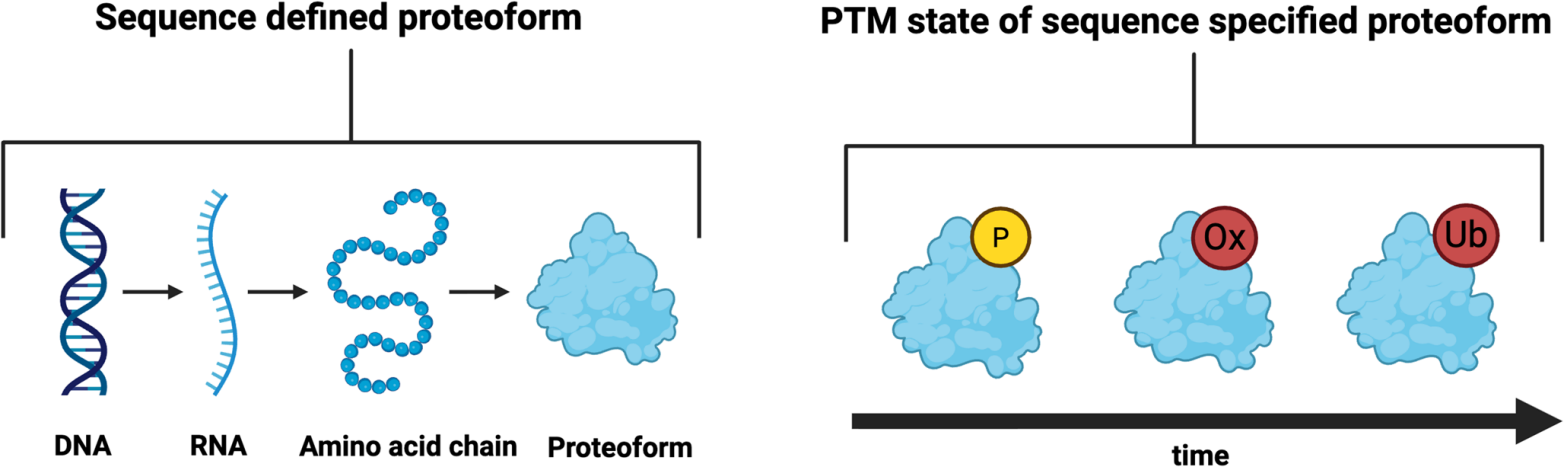
Proteoforms. Left. Proteoforms are first speciated at the level of their sequence by specific variants, from genetic alterations to splice isoforms. The result of this sequence variability is the production of specific proteoforms. Right. Once a sequence defined proteoform is produced, it is then modified over time by post-translational modifications (PTMs), such as phosphorylation, oxidation, and ubiquitination

Proteoform defines the identity of a protein molecule in a specific mode—a post-translational modification (PTM) *speciated* state (Fig. 1) (Siuti and Kelleher 2007; Jungblut et al. 2008; Tran et al. 2011; Smith et al. 2013, 2019; Aebersold et al. 2018; Smith and Kelleher 2018; Marx 2024).

An instantiated protein molecule has a specific amino acid (AA) sequence set by genetic variation and splicing (Venter et al. 2001; Auton et al. 2015). For example, one molecule derived from a single gene may bear a single nucleotide polymorphism (e.g., $\:G\to\:C\:at\:AA\:20$). Another, alternatively spliced molecule may lack the first exon (e.g., $\:AA:1-30$), yielding a truncated AA sequence with one fewer cysteine. Both molecules arise from the same gene, what marks them as distinct proteoforms is the speciated translation of mRNA to protein (Andersen et al. 2005).

From the moment of its birth during translation (Boisvert et al. 2007), a protein’s AA sequence is fixed until its molecular death—through cleavage, when one proteoform becomes two, or through degradation. During this lifetime, the “basis” proteoform—the molecule that departs the translational machinery—transforms into a series of self-similar but nonidentical modes via PTMs (Cobley 2025a).

In this way, the AA sequence serves as a canvas upon which the PTM-specified molecular portrait of the protein—its identity at any given moment—continuously evolves within a bounded possibility space. This possibility space encompasses every molecular portrait that can, in principle, be realised from that AA sequence—a landscape of potential selves constrained by structure, but open to transformation.

The molecule’s life is, therefore, not defined by a single form but by its evolution across forms (Carbonara et al. 2021). Function, too, must evolve. What we traditionally call “*protein function*” is in fact the outcome of a moving identity—the continuous expression of a molecule exploring its own possibility space (Cobley 2025a).

The protein basis of a phenotype is not a static protein or peptide but a functor of proteoforms—a mapping from molecular possibility to physiological effect. Life does not employ proteins as fixed tools; it draws upon ensembles of proteoforms that exist and interact as dynamic systems, each modulating the phenotype according to both its occupancy and functional weight within a perpetually evolving set. Proteoforms matter. They are not secondary decorations upon proteins; they are the information carrying operative units of biological reality—the true agents of protein function that link the proteome to physiology (Cobley 2024).

## The proteoform functor: mapping molecular identity to physiology

Each gene defines a sequence class, a set of possible primary structures arising from genetic variation and alternative splicing. Within each class, translation and post-translational chemistry generate an ensemble of realised molecular entities—the proteoforms. These belong to a state space $\:{S}_{i}$ ​describing all possible biochemical configurations—modes—accessible to the isoforms of gene $\:{G}_{i}$​.

Formally, a proteoform can be represented as a mode $\:{m}_{ij}\in\:{S}_{i}$​, where $\:j$ indexes the biochemical state defined by its specific set of PTMs. Transitions between modes defined by PTMs constitute morphisms $\:{\mu\:}_{jk}:{m}_{ij}\to\:{m}_{ik}$​. These morphisms capture the molecular dynamics by which one state evolves into another.

The proteoform functor, $\:F$, maps this molecular system onto its physiological realisation:

$$F:S_{i} \to \varphi _{i}$$

​where $\:{\varphi\:}_{i}$​ denotes the category of physiological functions associated with the proteoforms of gene $\:{G}_{i}$​. The functor preserves the structure of molecular transformations while translating them into biological outcomes. In categorical terms, if $\:{\mu\:}_{jk}$​ connects two molecular modes, then $\:F\left({\mu\:}_{jk}\right)$ connects their physiological manifestations.

Here, “physiology” is used in a deliberately broad and scale-inclusive sense to denote any functional consequence relevant to biological behaviour, spanning biochemical activity, cellular processes, tissue organization, and whole-organism phenotypes. Accordingly, enzyme activity is treated as an intermediate functional state within the functorial mapping rather than as physiology itself; physiological meaning emerges only as proteoform state transitions propagate across biological scales.

The full proteome can be conceptualised as a hierarchical mapping:

$$\:G\:\overrightarrow{\:translation}\,\, S\, \:\overrightarrow{F}\varphi\:$$

where $\:G$ is the category of gene-derived sequence variants, $\:S$ the category of proteoform state spaces, and $\:\varPhi\:$ the category of physiological effects. The composition of these mappings yields the organism’s realised biochemical identity—a continuous projection from genetic potential to physiological function.

Each protein’s life is an exploration of its local manifold $\:{S}_{i}$​, guided by biochemical morphisms and constrained by structure and environment. The proteoform functor $\:F$ transforms this exploration into physiology, preserving causality while translating scale. Hence, the function of a protein is not a fixed property but the image of its evolving state-space trajectory under $\:F$:

$$\:Function\:\left(t\right)=F\left({m}_{ij}\right(t\left)\right)$$

**Fig. 2:**
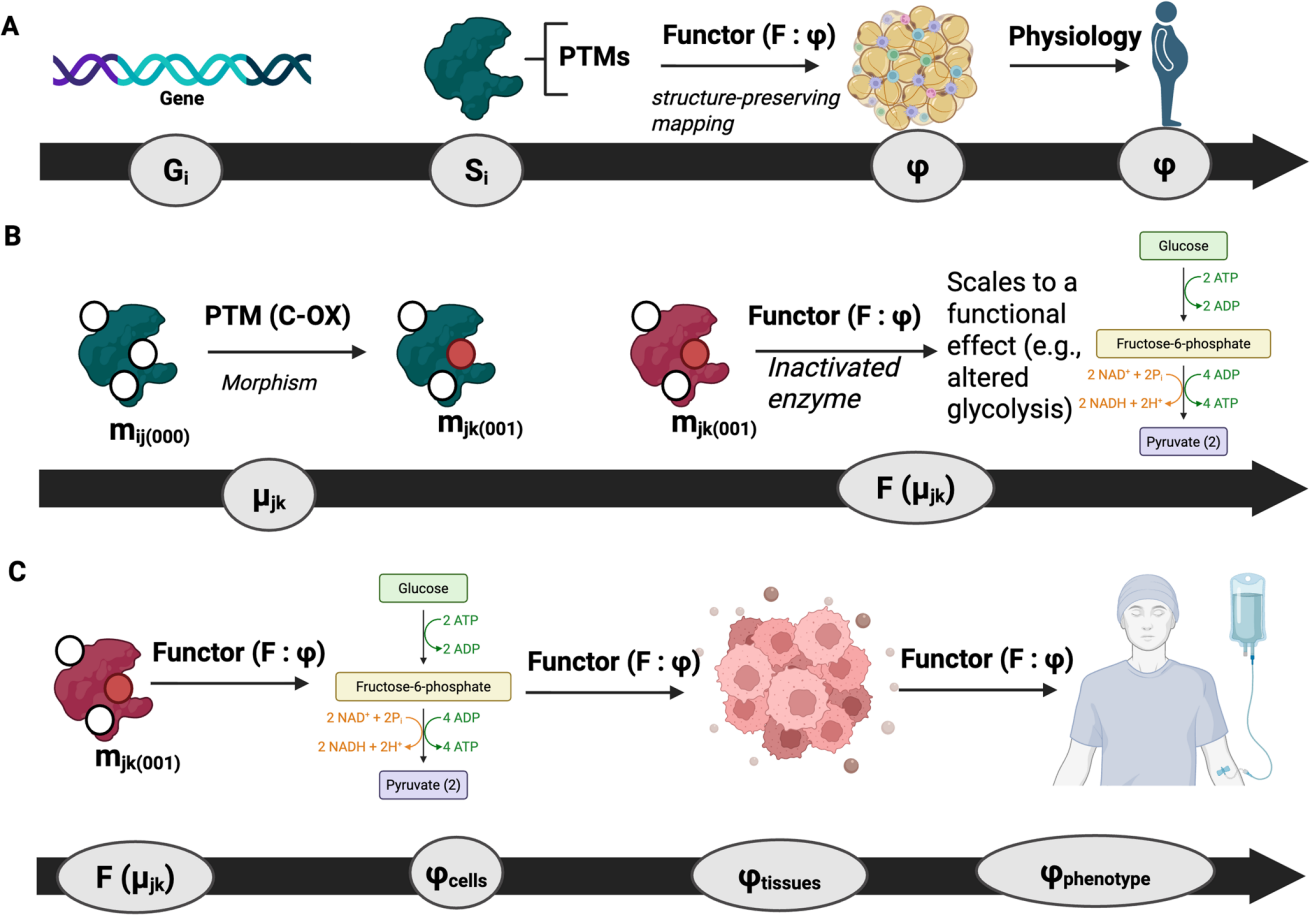
The proteoform functor maps molecular state transitions into multiscale physiological function. **A** Conceptual overview showing the mapping from gene-derived sequence classes (Gi) to proteoform state spaces (Si) via post-translational modifications (PTMs). The proteoform functor (F: S → Φ) acts as a structure-preserving mapping that projects molecular state transitions into functional domains across biological scales, culminating in physiological phenotypes (Φ). **B** Worked biochemical example illustrating a discrete proteoform morphism driven by cysteine oxidation (C-OX), transforming a reduced proteoform state (miⱼ(000)) into an oxidized state (mⱼₖ(001)). Application of the functor maps this molecular transition to a functional consequence (e.g., enzyme inactivation), yielding altered metabolic flux, illustrated here by perturbation of glycolysis. **C** Physiological scaling of functorial outputs: mapped proteoform transitions propagate from molecular function through cellular networks (Φ_cells_), tissue organization (Φ_tissues_), and ultimately to whole-organism phenotypic states (Φ_phenotype_), demonstrating how discrete proteoform dynamics give rise to emergent physiological behaviour.

This formalism provides a compact definition of molecular physiology: the emergent behaviour of a system of proteoform manifolds, each mapped to biological function through a structure-preserving functor (Cobley 2023a) (Fig. 2).

## Worked example: the proteoform functor in redox biology

Redox biology primarily deals with understanding how a set of small molecules, such as reactive oxygen species (ROS) like hydrogen peroxide (H_2_O_2_) (Winterbourn 2008; Dickinson and Chang 2011), influence complex phenotypes in health and disease (Murphy et al. 2011, 2022; Sies et al. 2022). ROS interface with a relatively small network of protein-based enzymes, such as superoxide dismutase (SOD) isoforms (McCord and Fridovich 1969; Imlay 2008, 2013), and albeit to a lesser extent most of the time the wider proteome (Go and Jones 2013; Go et al. 2015; Jones and Sies 2015; Moosmann 2021; Jones 2024). Electron exchange between ROS and the proteome—both the narrow redox enzyme set and the wider set of molecules—induce proteoform-level morphisms.

**Fig. 3:**
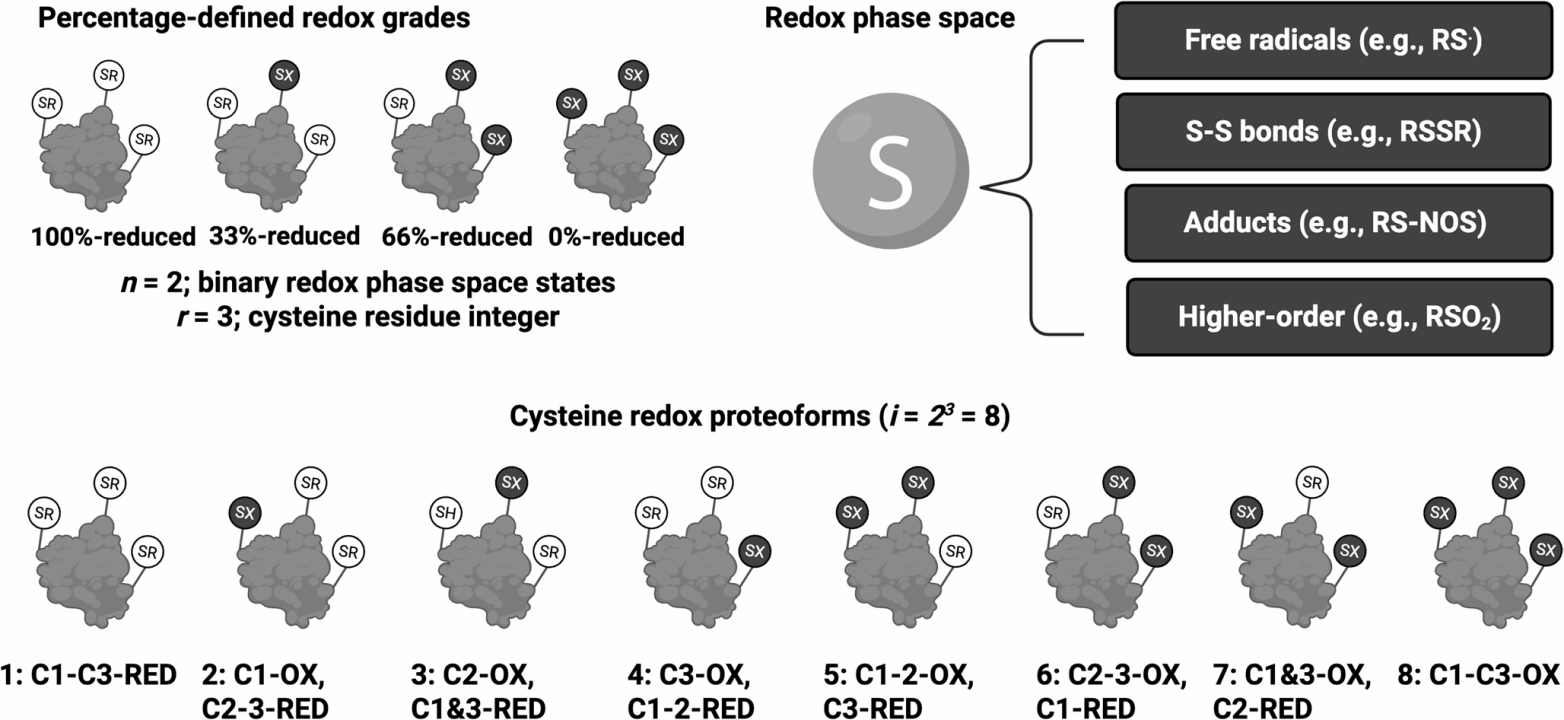
Cysteine proteoforms. Top left. Visual depiction of the quantized nature of cysteine proteoforms (see Box 2). The number of oxidised cysteines dictates the percentage oxidation of the molecule. Top right. Each oxidised cysteine occupies a specific post-translational state. For example, an oxidised cysteine might be part of an internal or external disulfide bond (RSSR). Bottom. The number of cysteine-proteoforms in binary for a protein with three cysteines

These proteoform-level morphisms include cysteine proteoforms (Cobley 2023b) (Fig. 3) Each cysteine-containing protein isoform $\:{G}_{i}$ defines a redox state space $\:{S}_{i}^{redox}$ comprising distinct modes per Modal Geometric Field (MGF) Theory (Cobley 2025a). For example, in simple binary reduced ($\:0$) and oxidised ($\:1$) terms (Cobley et al. 2025), a $\:{G}_{i}$ with two cysteines could adopt one of four modes: $\:{S}_{i}=\{00;\:10;01;\:11\}$ (Cobley et al. 2024a). Transitions between these quantized modes (Box 1) occur via redox reactions (Brown 2024; Cobley 2025b, c), represented as morphisms

$$\:{u}_{jk}:{m}_{ij}^{00}\to\:{m}_{ik}^{01}$$

These AA-specific (i.e., cysteine) modes define a manifold that belongs to the state space ($\:{S}_{i}$ ​) of that gene product ($\:{G}_{i}$). Applying the proteoform functor $\:{F}_{redox}$ to this system maps each mode and morphism to its physiological image:

$$\:{F}_{redox}:{S}_{i}^{redox}\to{\varphi\:}_{i}^{redox}$$

For example, for the human glycolytic enzyme GAPDH (three cysteines: 152,156,247), the morphism

$$\:{u}_{jk}:{m}_{ij}^{000}\to\:{m}_{ik}^{100}\:where\:1\:=\:Cys152-oxidised$$

Maps to the inactivation of the enzymes active-site (Peralta et al. 2015; Hyslop and Chaney 2022; Tuncay et al. 2023; Winterbourn et al. 2023). In turn, enzyme inactivation rewires metabolism, most commonly rerouting of glucose from glycolysis into the pentose phosphate pathway (Ralser et al. 2007; Talwar et al. 2023; Chatzinikolaou et al. 2024, 2025; Aburto et al. 2025).

Box 1 Quantized cysteine proteoform modes Cysteine proteoform modes are fundamentally discrete—they are quantized by their cysteine oxidation integer ($\:k$-state) (Cobley 2023a, 2024, 2025a; Cobley et al. 2024a, 2025). For example, a protein with three cysteines can form eight modes:

$$\:{S}_{i}=\{000;\:100;010;\:001;\:110;\:011;\:101;\:111\}$$

where each digit represents the redox state ($\:0$ = reduced, $\:1$ = oxidised) of a cysteine residue. These modes can then be grouped according to their $\:k$-state:

$$\:{k}_{0}=000$$

$$\:{k}_{1}=\:100,\:010,\:001$$

$$\:{k}_{2}=110,\:011,\:101$$

$$\:{k}_{3}=111$$

Like how electrons occupy discrete orbitals or how photons are absorbed at specific wavelengths, these *k*-states define allowed excitation levels in the manifold. The discreteness arises because there are no meaningful sub-integer intermediates between $\:0$ and $\:1$: the transition-state complex is represented by $\:1$, not $\:0.5$.

The number of ways each *k*-state can occur—its degeneracy—follows the binomial coefficients enumerated in Pascal’s triangle. For three cysteines, the degeneracy is $\:1\::\:3\::\:3\::\:1$. Thus, the relative population of modes naturally maps to quantized percentages (e.g., $\:0\%,\:33\%,\:66\%,\:100\%$-oxidised).

Importantly, *no individual molecule* with three cysteines can be “50%-oxidised” in the cysteine basis, though *a population* of molecules can collectively exhibit that ensemble average.

As a rule, the number of *k*-states equals the number of cysteines $\:\left(R\right)\:$plus one ($\:{k}_{max}=\:R+1$). Hence, the number of possible oxiforms—modes with at least one oxidised cysteine ($\:k=\ge\:1)$—is always the total number of modes minus one $\:({N}_{oxiforms}={S}_{i}-1)$.

The composite of these mappings defines the *functional topology* of redox regulation: local chemical transformations (morphisms in $\:S$) become causal influences on cell biology (morphisms in $\:\varPhi\:$) (D’Autréaux and Toledano 2007; Paulsen and Carroll 2013; Holmström and Finkel 2014; Alcock et al. 2017; Parvez et al. 2018; Sies and Jones 2020; Lennicke and Cochemé 2021a, b; Sies et al. 2024).

This mapping preserves the nonlinearity inherent to redox systems (Cobley et al. 2025). Small local changes can shift the occupancy of proteoform modes, altering the curvature of $\:{S}_{i}^{redox}$​ per MGF Theory and thereby reshaping its image in physiological space. Mathematically, a deformation in the molecular manifold induces a corresponding transformation in the physiological manifold:

$$\:{\delta\:\varphi\:}_{i}={F}_{redox}\left({\delta\:S}_{i}^{redox}\right)$$

In this way, cysteine oxidation becomes not merely a biochemical reaction but a mathematically formalised operator that bridges molecular chemistry and physiological function through a structure-preserving functor.

This structure-preserving functor matters physiologically (Cortese-Krott et al. 2017; Santolini et al. 2019; Feelisch et al. 2022). For instance, the redox basis of molecular exercise adaptations is often ascribed to changes—morphisms—in specific cysteine residues that define a unique oxidation pattern (Gomez-Cabrera et al. 2005, 2008a, b; Powers and Jackson 2008; Ristow et al. 2009; Nikolaidis et al. 2012; Cobley et al. 2015a, b, c, 2017; Margaritelis et al. 2016a, b; Cobley 2020b; Jackson et al. 2020; Margaritelis et al. 2020a, b, 2022, 2024; Nikolaidis et al. 2020; Williamson et al. 2020; Gomez-Cabrera et al. 2021; Cobley and Davison 2022a, b; Muggeridge et al. 2022; Cobley 2023a; Heaton et al. 2025). These transformations inevitably map back to proteoforms—the molecular entities that enact the phenotype. Despite their central importance, the identities of these proteoforms remain largely unknown.

This is not an isolated problem. Across physiology, the proteoform effectors of adaptation, signalling, and pathology are seldom identified—let alone quantified (Coorssen and Padula 2024). As a result, the field often describes processes in terms of aggregate protein changes rather than the molecular entities that truly define function. If the proteoform functor governs how molecular transformations map to physiology, then its experimental resolution is not optional—it is the missing link between biochemical potential and physiological reality. That is, the experimental parameterisation of the present conceptual framework is lacking, which reflects the limitations of current technologies and not the theory *per se*.

## Current proteomic technologies struggle to resolve proteoforms

***Bottom-up Mass Spectrometry (BU-MS)***

Although it is obligatory for functionally linking molecular biology—with its shifting biochemistry—to physiology, even state-of-the-art mass spectrometry (MS)-based technologies are limited in their ability to resolve proteoforms. Sophisticated peptide-centric workflows (Box 2) necessarily struggle to reconstruct the proteoform wholes from their peptide parts—a challenge likened to trying to put Humpty Dumpty back together again (Plubell et al. 2022). When a proteoform is fragmented into peptides, knowledge of its post-translationally speciated state is irretrievably lost. It is simply not possible to map all of the disembodied peptide parts back to the proteoforms that they once belonged to.

Box 2 Brief overview of BU-MS As a primer to more detailed guides (Aebersold and Mann 2003, 2016; Steen and Mann 2004; Sinha and Mann 2020), in BU-MS a sample comprising proteoforms is digested into peptides, usually using sequence-grade L-Trypsin to digest the proteoform at arginine $\:\left(R\right)\:$and lysine $\:\left(K\right)\:$residue.

In general, this produces short peptides, with most falling in the range of $\:5-25\:AA$, that bear a positive charge at their *N*-terminus, as well as, $\:R\:and\:K$ under acidic conditions $\:(pH\:\approx\:2\:to\:3)$. As a result, each peptide bears a mass $\:\left(m\right)$, given by the AA sequence and PTM state, and charge $\:\left(z\right)$—the product of positive minus negative charged groups.

Before the $\:m/z$ of the peptides is determined, they are separated using an online—physically coupled—high performance liquid chromatograph system. This involves the reverse-phased separation of peptides by their hydrophobicity on a carbon 18 (C_18_) column whereby more hydrophobic peptides elute later (rightward shift). Extremely hydrophilic peptides appear early in the gradient run with a gradual release of more hydrophobic peptides as the amount of organic solvent, normally acetonitrile, in the column increases.

Once eluted the peptides are converted into ions via electrospray ionisation (Fenn et al. 1989; Wilm et al. 1996; Jiang et al. 2020). These ion packets are then processed for MS^1^ and MS^2^ level analysis. In MS^1^, the $\:m/z$ of intact peptide ensembles is measured. After peptides are physically fragmented, the *m/z* of the resulting fragments is measured in MS^2^. Reading the m/z of the precursors (MS^1^—peptides) and their fragments (MS^2^—parts of peptides) enables sophisticated software packages to reconstruct the identity of the protein group (Cox and Mann 2008; Demichev et al. 2020; Lou et al. 2023; Frejno et al. 2025).

In some instances, specific modifications are studied (Larance and Lamond 2015). For example, in redox proteomics, light (reduced) and heavy (oxidised) labels are used to encode the redox state of the peptides, delivering information on the redox state of thousands of cysteine residues (Xiao et al. 2020). In general, PTMs are usually studied via enrichment to boost both the signal and thereby the coverage of the relevant sites (Day et al. 2021; Li et al. 2023; He et al. 2024). This involves separating peptides bearing the modification from the unmodified part of the sample, as in phospho-proteomics (Lancaster et al. 2024).

Operationally, BU-MS can be performed in either data-dependent acquisition (DDA) or data-independent acquisition (DIA) modes. Both schemes have their pros and cons (Cobley et al. 2019b). In general, DIA is preferred due to the increase depth of coverage and low level of data incompleteness, but the complexity of the fragmented spectra poses significant computational challenges (Tsou et al. 2015; Ludwig et al. 2018; Fröhlich et al. 2024; Welter et al. 2024; Kobayashi et al. 2025; Wen et al. 2025).

For example, detecting two peptide ensembles—one bearing a methionine sulfoxide and another bearing a phosphate group at serine—reveals that both modifications exist in the sample. Yet, it is typically impossible to determine whether these modifications coexist within the same proteoform or reside on distinct molecular species (Po and Eyers 2023). This ambiguity is amplified by the combinatorial explosion of possible modification states(Cobley et al. 2024a) and by incomplete sequence coverage (Timp and Timp 2020; Alfaro et al. 2021; Brady and Meyer 2022). Many database searches, constrained by computational limits, do not sample the full modification space, further fragmenting the picture.

As a result, current proteomic pipelines routinely collapse multiple isoforms and modified species into a single “protein group,” effectively averaging distinct proteoform populations into a single intensity value. The consequence is conceptual as much as technical: it erases the very molecular individuality that underpins function. Until proteomics can move beyond this reduction, the mapping between molecular identity and physiological consequence—the proteoform functor—remains unresolved (Carbonara et al. 2021; Coorssen and Padula 2024).

## Top-down MS (TD-MS)

TD-MS can, in principle, directly resolve proteoforms using high-resolution instrumentation (Roberts et al. 2024), such as Fourier-transform ion cyclotron resonance or Orbitrap-based systems (Scigelova et al. 2011). Measuring the intact mass of a protein species makes it theoretically possible to determine the $\:{\Delta\:}$-mass between its expected unmodified mass and its observed, PTM-inclusive mass (Ansong et al. 2013). This difference encodes the combined effect of modifications and sequence variants across all molecules derived from a given gene.

In practice, however, TD-MS faces multiple interrelated challenges that have limited its widespread adoption and interpretability:

1. *Sensitivity and spectral congestion*. When a protein population is divided into multiple proteoforms, the ion current is split across many mass/charge ($\:m/z$) bins in MS¹, reducing sensitivity for each species. In complex biological samples, overlapping charge states and isobaric species produce highly congested or chimeric spectra. Moreover, instrument resolution still favours proteins below $\:<60\:kDa$, excluding many physiologically relevant proteins like the ryanodine receptor (Place et al. 2015; Bellissimo et al. 2023; Nikolaienko et al. 2023).
2. *Combinatorial ambiguity*. Even when a distinct Δ-mass shift is observed at MS¹, the same shift can arise from many different combinations of PTMs or sequence variants. The resulting degeneracy creates a mapping problem between observed mass difference and true proteoform identity—analogous to observing the shadow of a shape without access to its geometry.
3. *Fragmentation limitations*. In MS², intact proteoform fragmentation is often incomplete (Kelleher et al. 1999; Siuti and Kelleher 2007; Chen et al. 2018; Brown et al. 2020). Chemical heterogeneity between proteoforms may alter bond dissociation patterns, producing variable or incomplete fragment coverage. Consequently, many spectra cannot unambiguously resolve which modification pattern produced the parent ion, particularly when PTMs may influence fragmentation energetics.

Together, these challenges constrain the current analytical capacity to identify and quantify proteoforms.

## Hidden cysteine proteoforms

The existential challenge of resolving proteoforms using current technologies is exemplified by cysteine proteoforms (Cobley 2024). As a physiological example of a wider trend: specific cysteine oxidation events are believed to underpin beneficial exercise adaptations (Henriquez-Olguin et al. 2020, 2023; Mason et al. 2020). While these oxidative events must map back to proteoforms—the distinct molecular entities that enact function—the identity of the relevant cysteine proteoforms remain elusive.

Although cysteine oxidation is frequently invoked to explain adaptive signalling, it is seldom measured directly (Kramer et al. 2018; Muggeridge et al. 2022; Tuncay et al. 2023), particularly in humans. In a rare example (Pugh et al. 2021), cysteine oxidation events were quantified by BU-MS in young and old human skeletal muscle following an exercise stimulus known to elicit redox-regulated adaptations (Cobley et al. 2012, 2013, 2014, 2016). However, due to the inherent limitations of BU-MS, the novel insights derived from this study could not be mapped back to the proteoforms responsible. Moreover, to the author’s knowledge, no TD-MS-based redox proteomic analysis of cysteine proteoforms in humans has yet been reported in an exercise context.

Even in this paradigmatic case—where ROS are proposed to mediate beneficial physiological effects—major gaps persist in the proteomic mapping of cysteine oxidation events. To date, only 154 cysteine oxidation sites have been quantified in human skeletal muscle both pre- and post-exercise (Stretton et al. 2020; Pugh et al. 2021), out of $\:\approx\:\mathrm{262,000}$ that comprise the human cysteine proteome. The identities of the specific proteoforms responsible for mediating redox regulation remain undefined.

Consequently, the redox functor that connects cysteine proteoforms to their physiological outcomes remains unmapped. The failure to resolve these mappings exemplifies how the absence of proteoform-level resolution breaks the causal correspondence between molecular identity and biological function. Until such mappings are made explicit, the mechanisms of redox regulation will remain fundamentally undetermined.

## Deriving the proteoform functor: what can be resolved now?

The formalism of the proteoform functor defines a complete mapping between molecular identity and physiology. In practice, however, this mapping can only be approximated from the limited observables accessible with current technologies. These current technologies do, however, enable vital information to be derived right now.

As recent genome-proteome mapping studies attest (Jakobson et al. 2025), it is now possible to identify and quantity the relative levels of the sequence variants that comprise the proteome. By combining different digestive proteases that cut at different AAs, most of the instantiated canonical splice variants in a proteome can be mapped (Sinitcyn et al. 2023). Likewise, elegant proteomic methods can map missense cysteine mutations (Desai et al. 2024).

When combined with personalised genome sequencing to provide a bespoke search space for the proteomic software (Consortium et al. 2020), comprehensive maps of the proteoform backbones—the AA sequences differentiated by PTMs—that do exist in a sample can be derived (Nagaraj et al. 2011; Kim et al. 2014; Thul et al. 2017; Bamberger et al. 2018; Seydel 2022). This vital information constrains what forms a given functor can take.

Estimations of the relative abundance of these proteins—no longer groups due to precise mapping—can be performed in parallel (Cox and Mann 2008; Nagaraj et al. 2011; Cox et al. 2014; Tyanova et al. 2016; Jiang et al. 2020; Ammar et al. 2023; He et al. 2024). When paired with reference standards to enable molar estimations (Malik et al. 2013; Wiśniewski et al. 2014; Welter et al. 2024), these analyses can mathematically cap the number of distinct PTM-speciated proteoforms that could exist in the sample. For example, protein copy number constrains the number of unique cysteine-proteoforms that could exist in a sample for most proteins (Cobley et al. 2025).

Since cysteine redox states and the relative abundance of protein groups can be measured simultaneously (Held 2020; Huang et al. 2023; Martinez-Val et al. 2025), deep sequence-resolved quantitative BU-MS analyses can infer the edge of the redox functor. Even when they cannot be explicitly mapped, near complete knowledge of all the cysteine redox states is a vital first step toward proteoform mappings using orthogonal technologies—proteoform-resolved immunoblotting (Renart et al. 1979; Towbin et al. 1979; Burnette 1981).

**Fig. 4:**
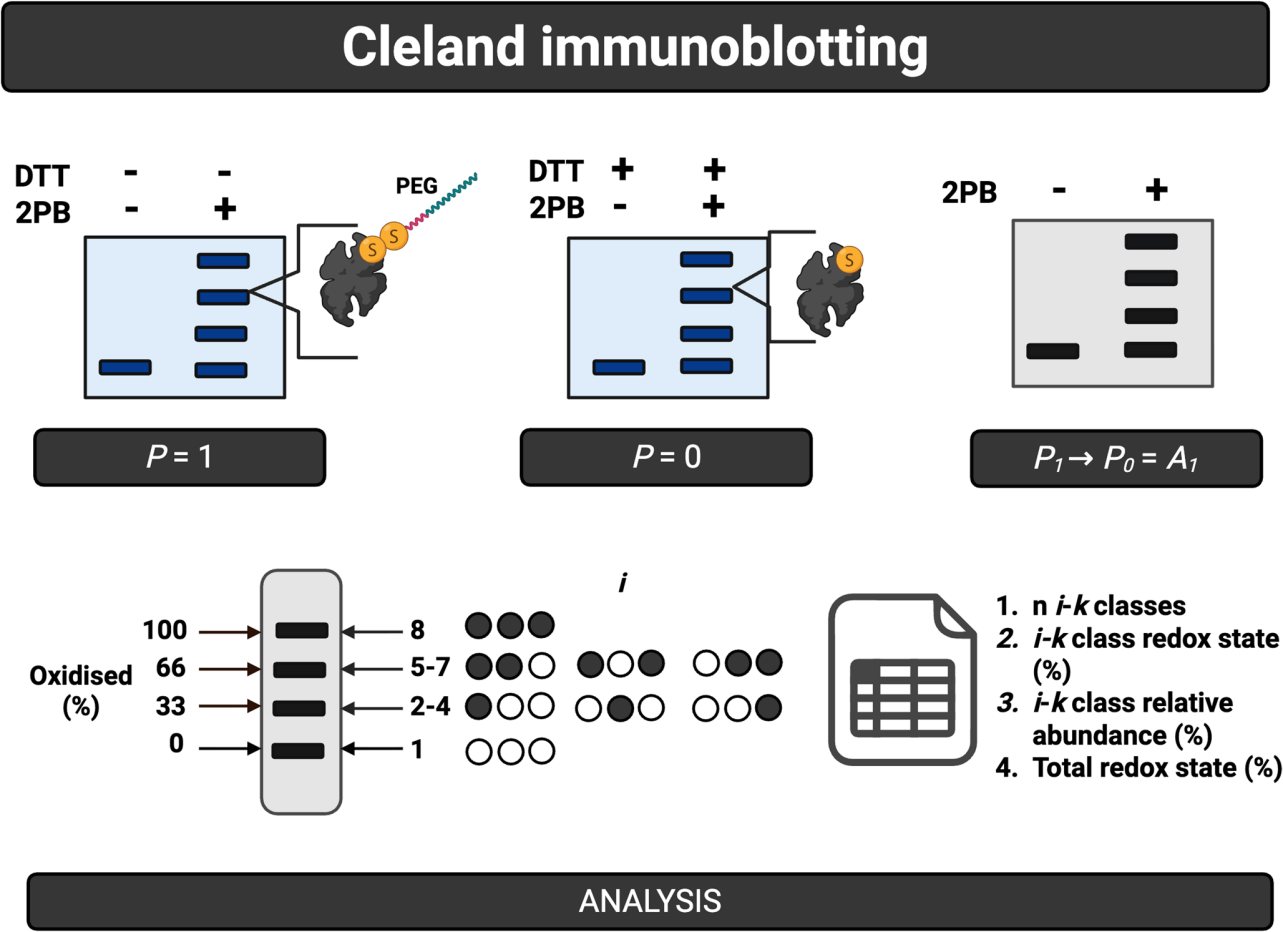
Cleland immunoblotting. In this workflow, oxidised cysteines are labelled with a reagent called 2PB, which contains a cysteine-reactive warhead, a PEG-payload and a biotin group. After the cysteine proteoforms have been resolved by nonreducing SDS-PAGE, the gel is treated with Cleland’s reagent: 1-4-dithiothreitol (DTT). DTT breaks the disulfide bond between the oxidised cysteine and 2PB, resulting in the removal of the PEG payload. Removing the PEG-payload enables the antibody to bind the different proteoforms separated by their *k*-state. As an example (bottom panel), all of the four *k*-states for a protein with three cysteines can be resolved. This enables the distribution of the cysteine proteoforms to be mapped

When paired with the BU-MS analyses of the same sample—the quantification of site marginals as the redox state of peptides, proteoform-resolved immunoblotting can map them to different $\:k$-states (Makmura et al. 2001; Burgoyne et al. 2013; Leeuwen et al. 2017; Cobley et al. 2019a; Lee and Chang 2019). The horizontal rows of Pascal’s triangle are projected as a series of vertical mobility-shifted bands, laddering the membrane by their $\:k$-state (Box 2). This $\:k$-state dependent mobility-shifted is induced by ectopic payloads, such as cysteine-reactive polyethylene glycol (PEG). The longstanding issue of these PEG-payloads sterically blocking antibody binding (Cobley and Husi 2020; Cobley et al. 2020), has recently been solved by Cleland immunoblotting (Cobley et al. 2024b, c). Cleland immunoblotting can, therefore, map site marginals to proteoform distributions (Fig. 4).

Together, these orthogonal measurements begin to define the measurable edge of the proteoform functor, providing the first direct map between the molecular manifold of cysteine oxidation and its physiological image. Consequently, even small-scale analyses can provide vital information on the proteoform functor, especially when combined with functional analyses, such as molecular pathway markers (Lennicke and Cochemé 2021a, b; Lennicke et al. 2025).

## Tackling the grand challenge: identifying and quantifying proteoforms at scale

The grand challenge now is to scale from partial, edge-level mappings to comprehensive, proteoform-resolved atlases of molecular physiology capable of deciphering complex phenotypes in humans. Partial information, while valuable, cannot resolve function because biology does not act on proteins—it acts through *proteoforms*. When molecular identity determines molecular fate, averaged or incomplete measurements obscure the true causal agents of physiology.

This limitation is exemplified by huntingtin (HTT) in neurodegeneration (Handsaker et al. 2025). Although total HTT abundance can be quantified using conventional peptide-centric proteomics, these measurements conflate multiple species that differ profoundly in length, structure, and toxicity. The disease-associated proteoform is a truncated exon 1 fragment containing the expanded polyglutamine repeat (Sathasivam et al. 2013). This fragment is mechanistically and clinically distinct from full-length HTT; it aggregates differently, interacts with different partners, and triggers unique downstream pathology. Measuring “*total HTT*” from shared, non-diagnostic peptides thus conflates healthy and pathogenic species, erasing the very information that defines disease. Both molecular understanding and biomarker discovery remain stalled until these proteoforms are explicitly resolved and quantified.

The same principle extends across biology. Nearly all lifestyle, environmental, and pharmacological interventions act by modulating proteoforms that remain unmapped—those that control catalytic activity, binding specificity, localisation, or turnover. For example, exercise combined with nutritional antioxidants acts on proteoforms (Forman et al. 2014; Cobley et al. 2015b; Halliwell and Gutteridge 2015; Forman and Zhang 2021; Halliwell 2023). Averaged measures of “protein expression” therefore risk describing *responses* rather than *mechanisms* (Cobley 2020a).

While comprehensively mapping proteoforms is challenging both conceptually and technically, advances in proteomic technologies are accelerating (Alfaro et al. 2021). Proof-of-concept studies support the potential for emerging nanopore and microscope-based technologies for the “long-read”, proteoform-resolved, digital measurement of intact molecules (Ginkel et al. 2018; Restrepo-Pérez et al. 2018; Swaminathan et al. 2018; Ouldali et al. 2020; Brinkerhoff et al. 2021; Egertson et al. 2021; Martin-Baniandres et al. 2023; Nova et al. 2023; Sauciuc et al. 2023; Wang et al. 2023; Yu et al. 2023; Filius et al. 2024; Li et al. 2024; Motone et al. 2024; Soni et al. 2025). By exploiting the logic of fluorescent microplate-based assays (Noble et al. 2021; Tuncay et al. 2022), many of these techniques can be adapted to cysteine proteoforms. While there are many challenges to overcome (MacCoss et al. 2023), these technological advances offer encouragement for addressing the grand challenge, especially when combined with advances in mass spectrometry (Brunner et al. 2022).

Regardless of the technology, defining the true identity of a proteoform functor will necessitate spatially resolved, such as organelle measurements, protein complex level, and single cell analyses (Bludau and Aebersold 2020; Eberwine et al. 2023; Gatto et al. 2023; Rosenberger et al. 2023; Hansen et al. 2024; Nordmann et al. 2024; Bubis et al. 2025; Guo et al. 2025). For example, the zonation of cysteine proteoforms in a tissue—their spatial distribution—provides insight into the proteoform functor than cannot be derived from bulk, aggregate measures alone. In this regard, their co-localisation on the outside of a tissue proximal to membranes may provide clues not only to their function but also production, such as via local entry of extracellular H_2_O_2_ into the cell via an aquaporin channel (Sies 2024).

## Conclusion

The capacity to resolve and quantify proteoforms defines the next frontier of molecular biology. By formalising the proteoform functor, a complete mapping can be envisioned between molecular identity and physiological consequence. In practice, this mapping remains incomplete, constrained by the limited observables accessible to current technologies. Yet these same technologies now define the measurable edge of the proteoform landscape, providing the first empirical foothold for a complete theory of molecular individuality.

Cysteine proteoforms exemplify this turning point. They expose the intrinsic nonlinearity of biological redox systems and the inadequacy of ensemble averages to explain function. Their discrete yet interconvertible modes reveal how information is encoded and transformed within proteins, bridging biochemical reaction space with physiological dynamics. Through this lens, oxidative events once described as stochastic can instead be understood as structured transitions within a higher-dimensional manifold.

Technological innovation is rapidly closing the gap between what can be measured and what must be known. Bottom-up proteomics, when paired with proteoform-resolved immunoblotting, already constrains the edge of the functor, while the advent of single-molecule, spatially-resolved, and “long-read” proteomic methods promises to extend it. The union of these approaches—experimental, computational, and theoretical—will allow the proteoform functor to be not only approximated but *solved*.

The implications extend beyond redox biology. Whether in ageing, exercise, or neurodegeneration, it is the proteoform—not the gene or the protein group—that determines phenotype. Mapping this layer of biological organisation transforms our understanding of life from a catalogue of molecular components to a dynamic geometry of identity. As this transition unfolds, proteoforms may emerge as the atoms of functional biology, the true units of physiological function.
